# Crystallographic and geochemical responses of giant clams on turbid reefs

**DOI:** 10.1038/s41598-025-90614-y

**Published:** 2025-03-02

**Authors:** Kimberley Mills, Sindia Sosdian, Duncan D. Muir, Eleanor H. John, Nadia Santodomingo, Kenneth Johnson, Ben Buse, Zarinah Waheed

**Affiliations:** 1 School of Earth and Environmental Sciences, Cardiff University, Cardiff, CF10 3AT UK; 2 Natural History Museum, London, UK; 3 School of Earth Sciences, University of Bristol, Bristol, UK; 4 Borneo Marine Research Institute, Universiti Malaysia Sabah, Kota Kinabalu, Malaysia

**Keywords:** Biogeochemistry, Environmental impact, Marine biology, Ocean sciences, Marine chemistry

## Abstract

Marine calcifying organisms on coral reefs face significant threats from various anthropogenic stressors. To better understand how these organisms will respond to a rapidly changing ocean, it is crucial to investigate their biomineralization across different reef environments. Despite their resilience and potential as conservation hotspots, turbid reefs—projected to expand throughout the 21st century—remain understudied, including a limited knowledge of biomineralization processes within these environments. Herein, for the first time, we assess the crystallographic and geochemical signatures of aragonite giant clam shells *Tridacna squamosa* from high and low turbid reefs in the Coral Triangle. Shell composition is strongly influenced by turbidity and biominerals formed in a high turbid reef show a more organized crystal orientation and significantly lower element-to-calcium ratios (magnesium/calcium, strontium/calcium). We hypothesize that these variations are driven by physiological changes related to the trophic flexibility of *T. squamosa*, utilizing both autotrophic and heterotrophic mechanisms. Observed differences may have implications for biomechanical and defense responses of shells, important in their ability to survive future change.

**Supplementary Information:**

The online version contains supplementary material available at 10.1038/s41598-025-90614-y.

## Introduction

Tropical coral reefs are highly vulnerable to the impacts of anthropogenic change^1^, already experiencing system-wide declines in many locations around the world due to large-scale coral bleaching from global warming, disease and overexploitation, and reduced water quality^2^. Comprehensive understanding of how reef building species make their biomineral skeletons in the face of future climate change is key to uncovering their sensitivity to changing oceanic conditions^3^. Turbid nearshore coral reefs represent over 12% of reefs globally and 30% of reefs in the Coral Triangle^4^. Turbidity is an optical parameter that represents the amount of light scattered and absorbed by suspended particles within the water column (e.g.,^5^). It may mitigate coral bleaching under elevated sea surface temperatures (SST) because the suspended particles reduce the amount of high irradiance reaching the reef organisms, thereby lessening the thermal stress associated with elevated irradiance levels^6–8^. However, marine calcifiers, including corals and giant clams, themselves enhance light scattering within their tissues, allowing them to optimize photosynthesis in low-light environments as well^9,10^. Given these opposing mechanisms, the overall effects of turbidity on the biomineralisation are ambiguous and require investigation. Further, it is crucial to understand skeletal formation processes and resilience in turbid reef environments because turbidity is forecast to increase throughout the 21st century due to anthropogenic activity, such as changes in land use, climate-driven increases in precipitation, resulting in greater run-off and higher sediment resuspension rates^11,12^.

Giant clams (Cardiidae: Tridacninae) are large and long-lived reef-dwelling bivalves that play key roles in reef ecosystems^13^. They are unique among bivalves in hosting zooxanthellae symbionts within their outer shell mantle, enabling them to carry out mixotrophic feeding, utilizing both autotrophic and heterotrophic mechanisms^14^. This symbiotic relationship, influenced by light-dependent physiological responses of the symbionts (family Symbiodinaceae) (e.g.,^15,16^), contributes to the clams’ sensitivity to environmental stressors, including mass bleaching events triggered by thermal stress^17,18^. Given their fast growth rates and long lifespans, giant clams are also extensively utilized as paleoenvironmental archives in the tropics^19^. However, interpreting changes in their shells in relation to their surrounding environment can be challenging. Like all biogenic carbonates, giant clam shells are influenced by both environmental factors and physiological adjustments governed by the organism^20^. This complexity underscores the importance of considering both the host’s physiology and symbiont activity when examining biomineral production. Additionally, environmental factors such as irradiance, food availability, and ontogeny, along with interspecific variation in photosynthetic performance, further influence their biomineralization processes^21^.

So far, work exploring differences in the balance between autotrophy and heterotrophy has shown that the fluted giant clam *Tridacna squamosa* can sustain similar rates of growth in high turbid and low turbid reefs, likely due to compensatory heterotrophic feeding under turbidity^21,22^. Relative contributions of autotrophy versus heterotrophy are particularly dynamic for *T. squamosa* from turbid environments with diverse food resources compared to other species of giant clam^22^, in similarity to corals with high mixotrophic capacity^23,24^. Yet, to understand if its biomineralization changes in response to turbidity, more information is needed, especially in relation to the properties of shell biominerals that are important for health and survival of the organism.

Microstructural and crystallographic arrangements (i.e., shell architecture) are the fundamental building blocks of skeletal organization and strongly influence shell biomechanical properties^11^. These features are known to vary in response to changing environmental conditions in many marine calcifiers, including corals and bivalves^25–29^. In giant clams, experimental studies have shown microstructural variation in relation to light^28^, warming and *p*CO_2_^30^, suggesting these parameters modify biomineralization pathways. In the natural world, an environmental gradient dictated by nutrient flux composed of phosphate, ammonium and nitrate has also been shown to be a factor in modifying daily to seasonal scale microstructure^31^. These nutrients impact the ability of tridacnids to mineralize their shells due to their consumption in support of photosynthesis, yet excess nitrogen and phosphorous significantly impact biomineral organization, revealing misshapen and porous biominerals^32,33^. Crystallographic properties are only known from studies of singular giant clam shells and show a strong preferred crystallographic orientation of the [001] axis and highly organized biomineral units^31,34^, contributing to the optimization of the organism’s protective shell capacity^34^. To date, however, it has not been investigated how shell microstructure and crystallography change across a natural environmental gradient, which offers insight into the ability of giant clams to make protective shells under changing oceanic conditions and identify the resilience of species.

The geochemical fingerprint of shells is thought to underpin biomineral design, associated crystallographic properties and their relationship to the environment because geochemical variations play a key role in the dictation of mineral formation^35^. Shell geochemistry shows a strong physiological component in many marine calcifiers because element-to-calcium (El/Ca) ratios deviate from surrounding ambient seawater (i.e., non-equilibrium fractionation) (e.g.,^36^). As with other bivalves (e.g.,^37^), giant clams show heterogenous composition of trace and minor elements including strontium (Sr) and magnesium (Mg) between^38^ and within their aragonite shell layers^39,40^. Even on subdaily temporal scales, there are large variations in El/Ca that are likely strongly related to diurnal cyclicity and inherent biological rhythm^40^. Mg/Ca and Sr/Ca are at current particularly difficult to interpret because they are hypothesized to be under strong biological control^39–45^. Mg is highly associated with the organic components of the shell (e.g.,^46^) and is often substantially elevated near the organic-rich daily growth lines in giant clams, revealing daily Mg cyclicity (e.g.,^39^). Similarly, Sr cyclicity is observed at a daily scale, but is instead associated with active transport mechanisms (e.g., Ca-ATPase) at mantle epithelia running on a diurnal cycle^41,43^.

To better understand the fundamental mechanisms of biomineralization in giant clams, and how shell construction changes with environmental conditions, we investigate the distribution of microstructure, crystallographic texture and element-to-calcium ratios (Mg/Ca; Sr/Ca) of biominerals in mixotrophic fluted giant clams *Tridacna squamosa*. Firstly, we assess crystallo-chemical variation at the intra-shell scale by analyzing different shell layers (inner shell layer, outer shell layer and the myostracum, involved in shell muscle attachment). Secondly, we compare features between shells collected from high turbid and low turbid coral reefs in the Coral Triangle region of Northern Borneo to understand the impact of turbidity on biomineralization. The high turbid reef (Triangle reef) is approximately 3.5 km from a river mouth, discharging sediment to the site at low tide, while the low turbid reef (Baik reef) has low sediment input and activities at the site include recreational diving. We provide insight into the potential role of the environment and physiology on (1) the giant clam host and (2) associated photosymbionts, with a focus on relative contributions from mixotrophy. Overall, this study has major implications for how giant clams build their protective shells under changing oceans and pinpointing plasticity in biomineralization responses.

## Results

### Microstructural features

**Fig. 1:**
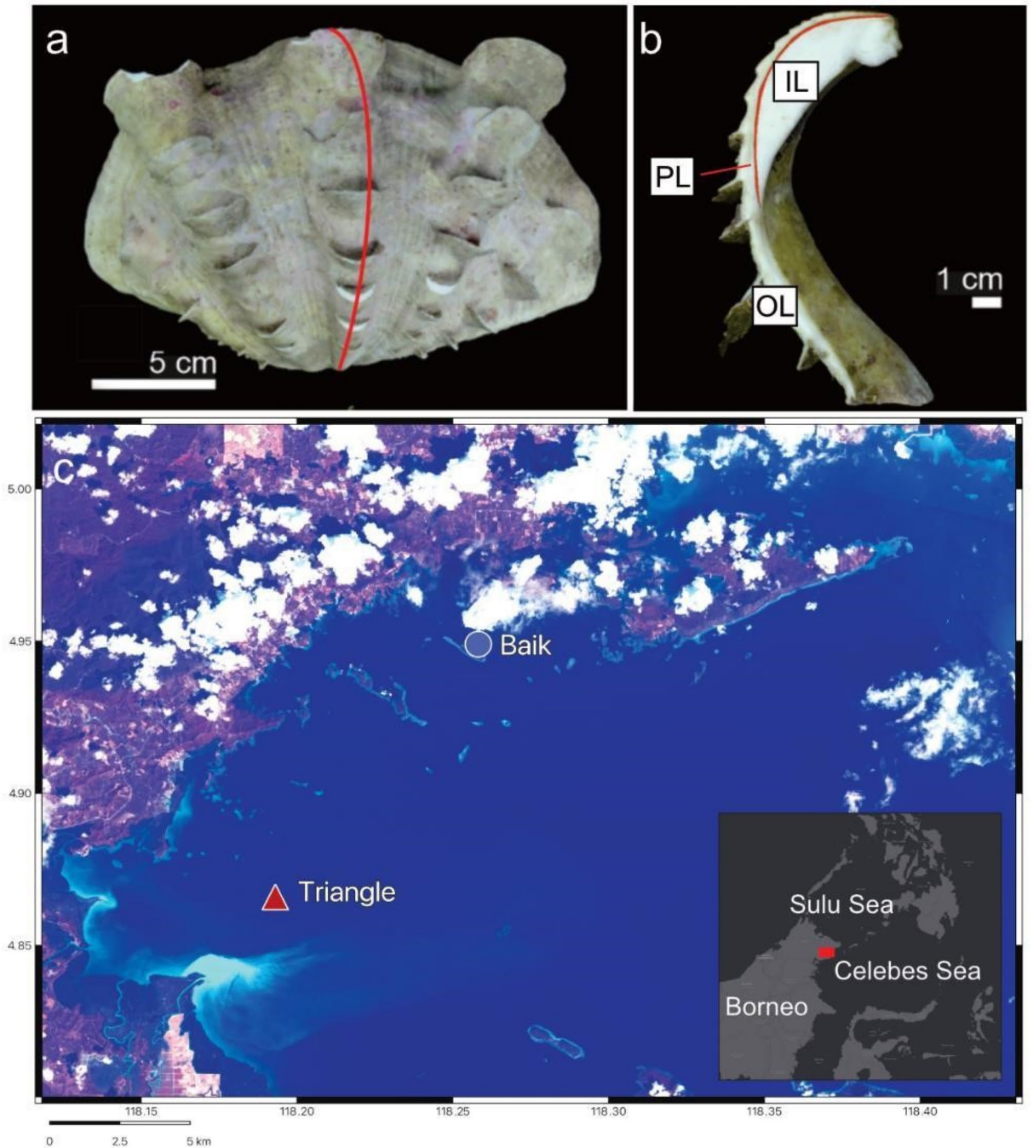
Sample collection localities of giant clam shells. (**a**) Valve of *Tridacna squamosa* with red vertical line indicating location of cut slice. (**b**) Longitudinal slice taken from (**a**), highlighting the inner layer (IL), outer layer (OL) and pallial line (PL). (**c**) False colour composite map (bands 7/4/2) of Triangle reef (high turbid) and Baik reef (low turbid) sample sites. River plume originating from the Tingkayu river highlighted in bright blue near Triangle reef.

This research investigated the microstructure of four live collected *T. squamosa* shells in Darvel Bay (4° 5356′ N, 118° 2646′ E) on the eastern coast of Sabah, within the Coral Triangle region of eastern Malaysia (Fig. 1). Specimens were collected in 2019 and 2020 from two distinct reef environments: Triangle reef, high turbid and near the mouth of the Tingkayu River and 2. Baik reef, low turbid (Fig. 1, Supplementary Table 1). Two of the four specimens collected had a comparable lifespan of around 3 years^16^ (Supplementary Table 1) and were collected at each site one day apart, allowing for direct comparison. We identified micron-scale skeletal structures with field emission gun scanning electron microscopy (FEG-SEM) in specimens collected from the two reef sites.

**Fig. 2:**
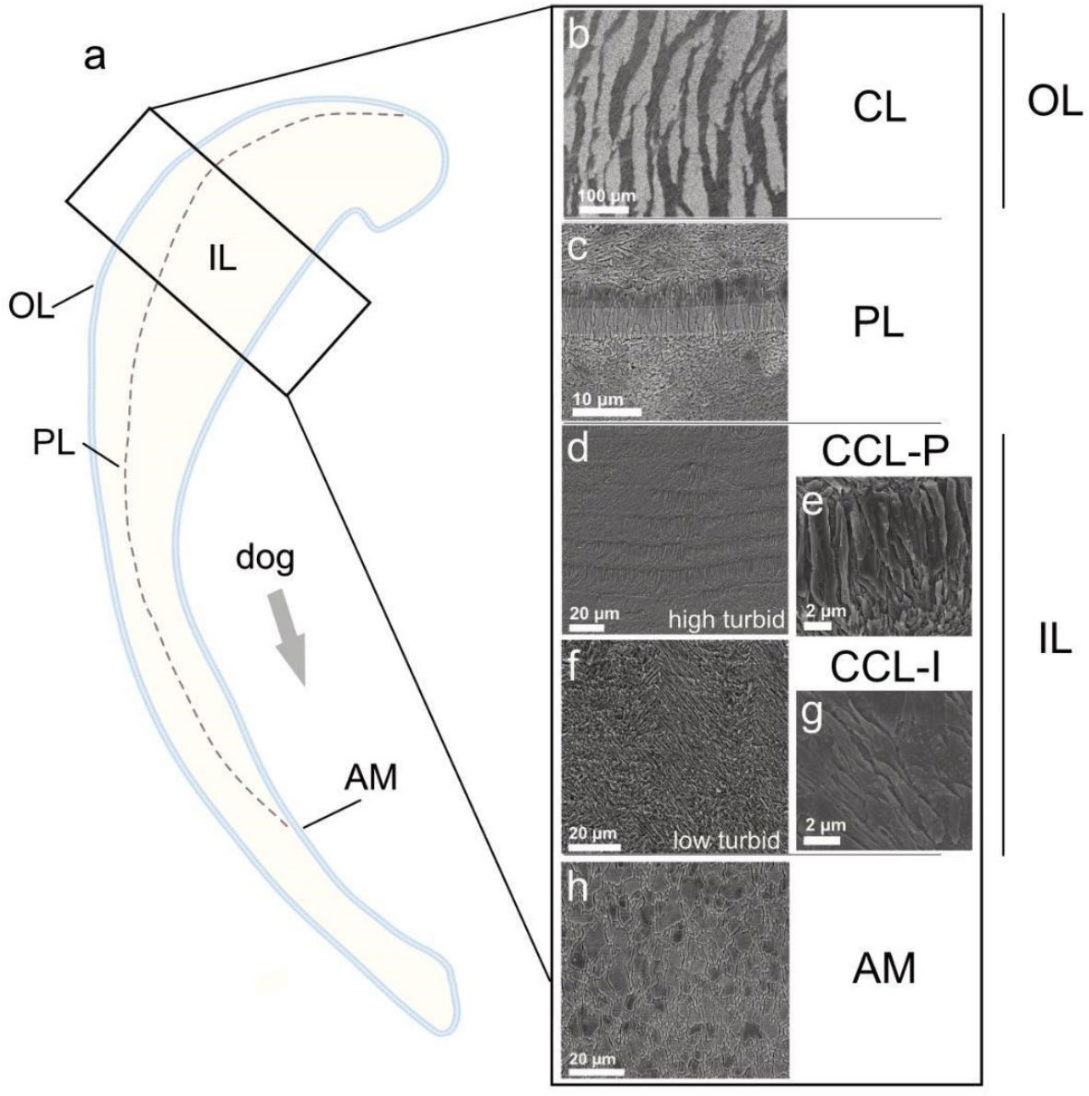
Microstructure of high turbid and low turbid giant clam shells. (**a**) Schematic longitudinal slice of the shell of *Tridacna squamosa* highlighting the inner layer (IL), outer layer (OL), pallial line (PL) and adductor myostracum (AM). Black rectangle denotes location of analysis for the IL, OL and PL. (**b**–**h**) SEM images of the different layers and associated microstructures throughout high turbid (Triangle reef) and low turbid (Baik reef) shells: (**b**) crossed lamellar microstructure (CL) in the OL; (**c**) PL separating the IL and OL; (**d**,**e**) complex crossed lamellar microstructure with prisms (CCL-P) in high turbid shells; (**f**,**g**) irregular complex crossed lamellar microstructure (CCL-I) in low turbid shells; (**h**) rounded polygonal prisms of the AM.

Giant clams have a two layered shell, consisting of an inner shell layer (IL) and outer shell layer (OL), demarcated by the pallial myostracum (or pallial line) (Figs. 1a,b and 2). Identified aragonitic microstructures from the OL to IL in *T. squamosa* consisted of crossed-lamellar (CL) (Fig. 2b), irregular complex crossed-lamellar intersected with simple irregular or regular prisms (CCL-P) (Fig. 2d,e) and irregular complex crossed-lamellar with an absence of prisms (CCL-I) (Fig. 2f,g).

The outer layer, composed of crossed-lamellar (Fig. 2b), was identical in all shells regardless of collection locality. The three hierarchical structural orders of lamellae in this microstructure were markedly clear: first-order lamellar, which in turn comprised second-order lamellae, made up of nanoscale third-order biomineral units^35,47–49^.

In contrast, the inner layer consisted of complex crossed lamellar (Fig. 2d–g) that showed fine-scale skeletal variation among reefs. Two regular morphologies of daily growth increments that intersected lamellae were defined: (1) CCL-P in high turbid Triangle reef, composed of frequent irregular or regular simple prisms coupled with a thinner layer of smaller, oblique crystallites^22,31^, varying in length and masking the underlying microstructure (Fig. 2d,e); (2) CCL-I in low turbid Baik, composed of adjacent growth lines cutting through the microstructure (Fig. 2f,g). Shells collected from the Triangle reef showed CCL-P in latter shell regions only (i.e., mid shell to inner layer edge) and CCL-I in juvenile portions of the shell adjacent to the pallial myostracum, suggesting an age-related influence.

Myostraca are sites of attachment for bivalve muscle soft tissues to the mineral shell surface^49^ and separate the inner and outer layers in giant clams. The pallial myostracum at the studied shell region (~ 4 mm along from the hinge) comprised elongate regular simple prisms (Fig. 2c). Bivalves also have adductor and pedal retractor myostraca, of which the former is a local thickening of the pallial myostracum^50^. When traced from the umbo to the upper shell margin, the width of the pallial myostracum progressively increased and crystal morphology changed, with single rows of elongate prisms modified to rounded polygonal sheet-like rows of prisms of the adductor myostracum (Fig. 2h).

Noteworthy, at higher resolution, a nanoscale composite granular assembly typical of amorphous calcium carbonate precursor phases of biomineralization^51,52^ covered the surface of biominerals across shells. Nanoparticles ~ 80–200 nm in size with an irregular rounded shape were present in all microstructures.

### Crystallographic features

**Fig. 3:**
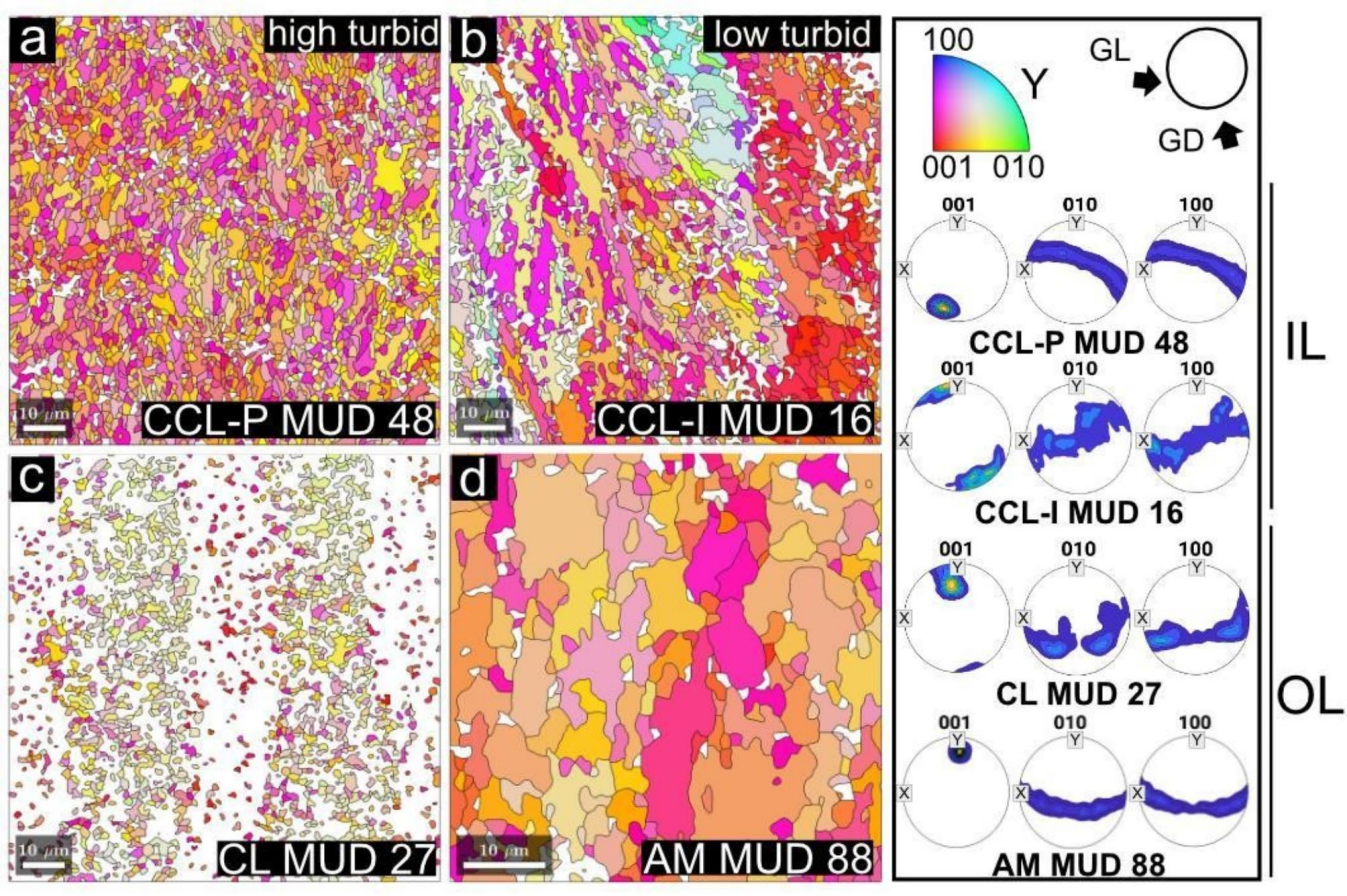
Crystallographic texture of high turbid and low turbid giant clam shells. EBSD inverse pole figure maps (IPF-Y) and corresponding contoured pole figures of different layers and associated microstructures in high (Triangle) and low (Baik) turbid giant clam shells. (**a**) Aragonitic arrangements of complex crossed lamellar microstructure with prisms in the IL of high turbid shells (MUD 48). (**b**) Irregular complex crossed lamellar microstructure with lower crystallographic orderliness (MUD 16) in the IL of low turbid shells. Identical arrangements of (**c**) crossed lamellar (MUD 27) and (**d**) adductor myostracum (MUD 88) present in the OL of both high and low turbid shells. Contoured pole figures show a common preferred crystallographic orientation of the [001] axis in all identified microstructures. The MUD values associated with each pole figure indicate different co-orientation strengths between the IL in high and low turbid shells, as well as between different regions of the shell. Reference pole figure shows orientation of GL (growth lines) and GD (growth direction).

We use Electron backscatter diffraction (EBSD), a SEM-based technique involving electrons diffracted along lattice planes of a sample’s crystals, which provides quantitative information on crystalline structures and orientation of each grain. The crystallographic texture of the *T. squamosa* shells revealed a coherent crystallographic preferred orientation of the aragonite [001] axis in all samples (*n* = 4), showing one strong pole figure maximum (Fig. 3). The studied shells showed high variation in multiple of uniform density (MUD) values, which indicate crystallographic co- orientation across the different microstructures. The crossed-lamellar microstructure associated with the outer layer had MUD values between 27 and 30 (Fig. 3c). Sets of alternating first-order lamellae ~ 15–30 μm wide were apparent and showed a belt-like distribution of the [100] axis and [010] axis around the [001] axis. However, we caution the low diffraction quality in this region and fewer aragonite points were indexed (Fig. 3c) compared to the IL (Fig. 3a,b), likely related to its smaller grain size (~ 1 μm).

The IL had larger grain sizes (above ~ 1 μm) with stronger diffraction and higher indexing of aragonite points. Overall, MUD values in the IL varied between 16 and 48 (Fig. 3a,b). Areas of first shell growth adjacent to the pallial line with a CCL-I arrangement shared microtextural continuity with the OL (i.e., lamellae carry over from the OL) (Fig. 3b). Away from the pallial line and OL, a stronger intensity of fabric developed in shells from Triangle that showed the CCL-P arrangement (Fig. 3a), with a clear one-dimensional orientational order, where [100] and [010] axes formed a continuous girdle around the [001] axis. CCL-I had a wider belt-like distribution of the [100] and [010] axes and more scattered [001] maxima. Accordingly, co-orientation strength between CCL-I and CCL-P showed MUD values of 16–24 and 45–48 respectively. The adductor myostracum (Fig. 3d), imaged further up the shell section by tracing the shell along from the hinge nearer to the ventral margin had the largest grain sizes (~ 5–20 μm) and highest co-orientation strength of MUD 88.

### Geochemical maps

**Fig. 4:**
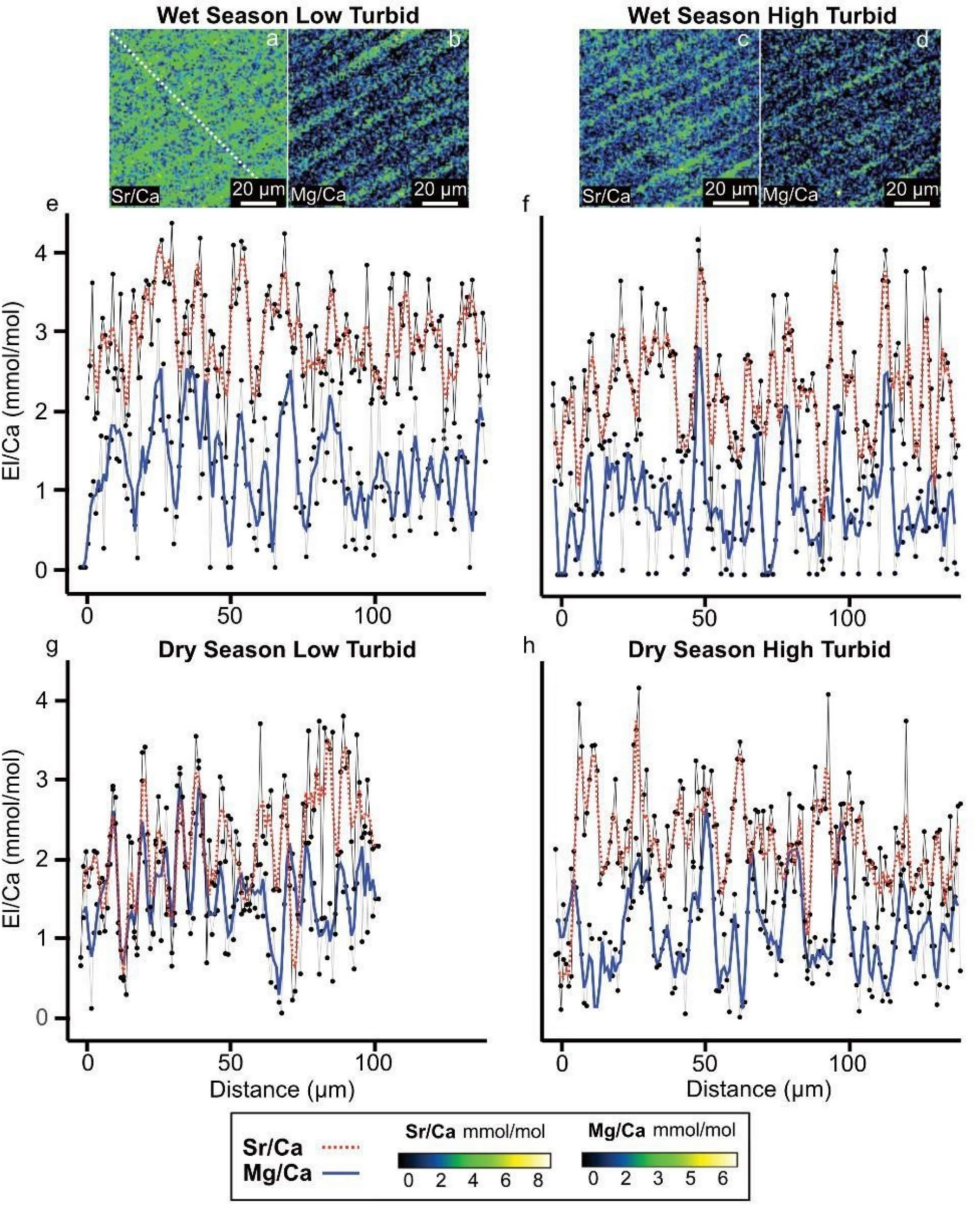
Geochemical profiles of element-to-calcium ratios for high turbid and low turbid giant clam shells. (**a**–**d**) Sub-daily resolved EPMA maps for the inner shell layer of low turbid (Triangle) and high turbid (Baik) giant clams within a region of shell growth corresponding to the wet season. White dashed diagonal line in (**a**) denotes transect measurement locations of maps for corresponding profiles for Sr/Ca and Mg/Ca shown in (**e**–**h**). (**e**) Sr/Ca and Mg/Ca profiles for a portion of growth in the low turbid shell collected in 2019 corresponding to the wet season. (**f**) Portion of growth in the high turbid shell collected in 2019 corresponding to the wet season. (**g**) Portion of growth in the low turbid shell corresponding to the dry season. (**h**) Portion of growth of the high turbid shell corresponding to the dry season. Color bars below profiles show concentrations of element-to-calcium ratios in mmol/mol corresponding to maps in (**a**–**d**). Grey lines on profiles show raw EPMA data. Red dashed (Sr/Ca) and solid blue lines (Mg/Ca) give a 3-point rolling mean.

We used electron probe microanalysis (EPMA) to investigate Mg/Ca and Sr/Ca ratios of shells collected from high and low turbid reefs on a comparable scale to microstructural and crystallographic data, specifically examining wet and dry seasons in the IL and OL. In the IL, thin (~ 2–8 μm) high Mg and Sr banding revealed daily periodicity, cohering with daily growth increments (Fig. 4a–d). Mean Mg/Ca and Sr/Ca ratios taken from line transects (white dashed line on Fig. 4a indicates location) between high turbid and low turbid reefs varied from 0.90 ± 0.55 to 1.56 ± 0.66 mmol/mol and 2.09 ± 0.70 to 2.95 ± 0.58 mmol/mol respectively (Fig. 4e–h). In the outer layer, collection of data from the high turbid reef was unsuccessful due to extensive microboring of the shell surface. However, this region in the low turbid reef showed no alteration and mean Mg/Ca and Sr/Ca ratios taken from a transect were similar to the inner layer (1.42 ± 1.29 and 2.09 ± 1.20 respectively), with high Mg and Sr bands presumably related to daily growth increments (Supplementary Fig. 2).

Our focus was on element-to-calcium ratios measured from regions of shell growth that corresponded to the wet- and dry seasons in our study locality. At an intershell level, median Mg/Ca from the low turbid shell was significantly elevated in the dry- and wet season (Kruskal–Wallis, χ^2^(3) = 40.7, *P* < 0.05, pairwise comparisons with Dunn’s multiple comparisons test) compared to the high turbid shell, while mean Sr/Ca from the low turbid shell was significantly elevated in the wet season only (one-way ANOVA, *F*(3) = 43.58, *P* < 0.01, pairwise comparisons with Tukey HSD test) (Supplementary Tables 2 and 3). Within the same shell, Sr/Ca and Mg/Ca from the low turbid shell significantly varied between seasons (one-way ANOVA, *F*(3) = 43.58, *P* < 0.01, pairwise comparisons with Tukey HSD test; Kruskal–Wallis, χ^2^(3) = 40.7, *P* < 0.05, pairwise comparisons with Dunn’s multiple comparisons test, respectively), while the high turbid shell showed no significant difference in Sr/Ca or Mg/Ca (one-way ANOVA, *F*(3) = 43.58, *P* > 0.05, pairwise comparisons with Tukey HSD test; Kruskal–Wallis, χ^2^(3) = 40.7, *P* > 0.05, pairwise comparisons with Dunn’s multiple comparisons test, respectively) (Supplementary Tables 2 and 3). However, we do acknowledge the uncertainties of interpreting this data on a seasonal scale because of the small areas of shells sampled with EPMA, which is a trade off with the high spatial resolution of the data.

## Discussion

The findings of this study show that the giant clam *Tridacna squamosa*, an important reef-dwelling bivalve from both an ecological and palaeoceanographic perspective, exhibits plasticity in biomineralization pathways between high turbid and low turbid coral reefs. For the first time, we highlight fine-scale variations in the microstructure, crystallographic organization and minor and trace element geochemistry of shell biominerals, which are not resolvable at coarser sampling resolutions. We find that shells formed in the high turbid reef situated near a river source have a different shell microstructure in the inner shell layer, consisting of paired daily growth increments with higher crystallographic co-orientation and lower Mg/Ca and Sr/Ca, compared to lower crystallographic coorientation and higher Mg/Ca and Sr/Ca in the low turbid reef. Key environmental differences between the two reef localities are *K*_*d*_(490), total suspended solids and chlorophyll-*a*, which are elevated in the high turbid reef^16^. In a previous study^16^, we hypothesized that elevated concentrations of these parameters year-round may suggest *T. squamosa* can utilize different relative contributions of feeding type to total organismal energy requirements^22^. These environmentally driven changes to physiological machinery may imprint on its biomineralization pathways and thence architecture and geochemistry of shells. This is the first study to show that differences exist in the biomineralization of giant clams on turbid reefs, which is important because knowledge of giant clam physiological responses to different anthropogenic stressors is vital for conservation strategies moving forward under a rapidly changing ocean^53^.

**Fig. 5:**
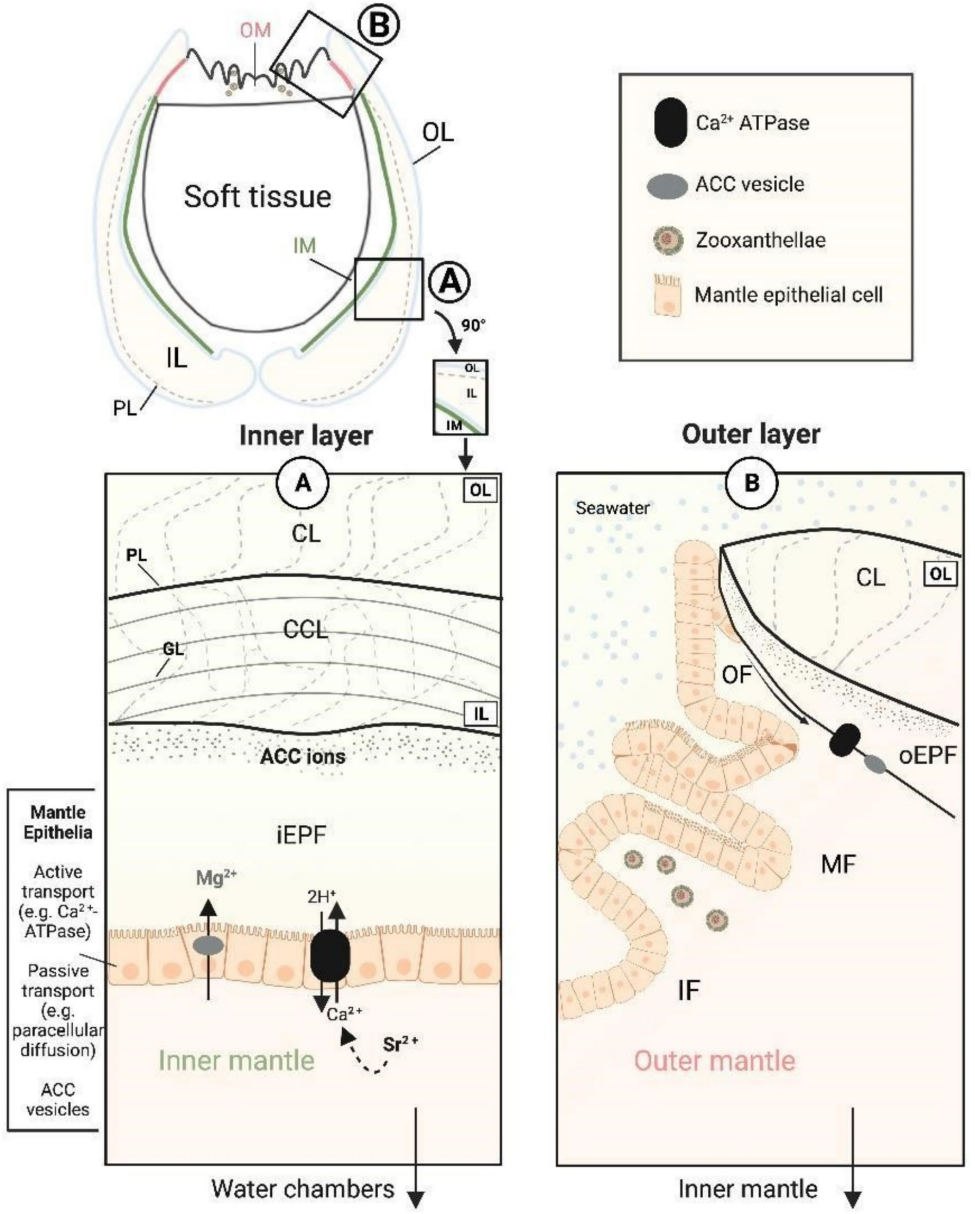
Conceptual representation of biomineralization in giant clams. Not to scale. Shell mineralization pathways for each shell layer in relation to the inner shell mantle (IM) and outer shell mantle (OM) shown. Formation of the shell (Ca^2^ + HCO_3_^−^ → CaCO_3_ + H^+^) takes place in an extracellular privileged space, known as the extrapallial fluid (EPF) (e.g.^57^). The inner shell layer (IL), with a complex crossed lamellar microstructure (CCL) (**a**) forms from the inner EPF (iEPF) and the outer shell layer (OL), with a crossed lamellar microstructure (CL) (**b**) from the outer EPF (oPF). The IL and OL are demarcated by the pallial line (PL). Transport of Ca to the EPF occurs at the mantle epithelium through active (e.g., Ca^2+^ ATP-ases)^52^ and passive (e.g., paracellular diffusion) transport mechanisms^3^. Organic and Mg rich amorphous calcium carbonate (ACC) vesicles cross the epithelial cell membrane and are transported to the calcification front. ACC particle attachment and subsequent ion attachment occur at the biomineral growth front and crystallize into aragonite (e.g.,^58^). Zooxanthellae are housed extracellularly in Z-tubules (not shown) within the inner fold (IF) of the OM and carry out photosynthesis from sunlight to provide nutrition to the clam through an autotrophic feeding pathway^54^. The EPF is partially open to surrounding seawater but is chemically different from seawater due to ‘vital effects’ tightly controlled by the organism^17^. The IL is situated between the IM and OL, in close contact with the iEPF and physically further away from seawater (**a**). The OL is in direct contact with seawater, where OM specific mechanisms are carried out and the clam undergoes light-dependent physiological phenomena^18^. In the case of Mg and Sr, different predominant pathways are responsible for transportation to the site of calcification (**a**). Mg is associated with the organic or ACC phase because its ionic radius is not favorably substituted in the orthorhombic crystal lattice of aragonite^44,55^. Sr is substituted for Ca within the aragonite lattice due to similar ionic radii and may predominantly enter the EPF through active transport mechanisms such as Ca^2+^ ATPase (e.g.,^18^).

The microstructural changes we identified for *T. squamosa* display variation of daily increments within the inner shell layer (IL) between reefs, with irregular complex crossed-lamellar (CCL-I) in the low turbid reef and paired daily growth couplets with a thinner layer of small crystals and larger prisms (CCL-P)^15,22,31^ in the high turbid reef. Both microstructures show a common preferred crystallographic orientation of the [001] axes roughly perpendicular to growth increments and parallel to the growth direction, similar to previous EBSD studies of *Tridacna gigas*^31^ and *Tridacna derasa*^34^. However, crystal co- orientation, or organization, measuring how orientated crystals are relative to one another, is higher for CCL-P (MUD 45–48) compared to CCL-I (MUD 16–24). This is higher than other tridacnids^34^, but within the realm of other bivalves with similar microstructural arrangements, such as *Glycmeris glycmeris*^54^. However, despite this variability in the IL, the OL and myostracum microstructures are identical in all shells we examined from both reef sites. The myostracum is known to be highly conserved and universal across most of the phylum Mollusca^49^ and may either grow homoepitactically on top of the OL^54^ or directly attach to underlying muscle fibers by adhesive epithelial cells^55^. While our results for the OL concur with a previous study^31^, which showed that *T. gigas* has an identical microstructure between different reef sites, the authors suggested that the OL is under tight biological control due to a similar mineralization mechanism across individuals. However, we offer an alternative hypothesis based on our observations of fine-scale variation in the IL—the IL is under high biological control, regulated by complex physiological machinery that is reflected in microstructure. This may be underpinned by: (1) The IL is situated further away from the external environment and may be less open to seawater (Fig. 5a); (2) The inner mantle is always in direct contact with the extrapallial fluid in the IL compared to the outer mantle^56^ (Fig. 5a,b); (3) Mantle epithelial cells lining the IL may be more tightly packed than the OL^55^, which could minimize paracellular diffusion and leakage. Therefore, it may be that the physical location of the IL and its distance to surrounding seawater is important in the determination of the degree of physiological control.

The outlined variations observed in the architecture of the IL are reflected in the elemental distribution of shells. Concentrations of El/Ca shown in the EPMA maps reveal that in the low turbid reef with CCL-I microstructure, there is significantly higher Mg/Ca compared to the more turbid reef with CCLP. On a daily scale, Mg/Ca is elevated at the boundary between daily growth lines regardless of reef environment. In biogenic aragonite, Mg is associated with the organic or highly disordered amorphous calcium carbonate (ACC) phase because its small cationic radius does not favor incorporation into the orthorhombic structure of aragonite^44,55^ (Fig. 5a). Although shell organics represent a small portion of total shell weight equating to ~ 1% in giant clams^34^, their presence and heterogenous nature likely plays an important role in relation to the concentration of Mg/Ca. For instance, elevated Mg/Ca is linked to increased organic contents at the boundary between growth lines in many aragonitic bivalves (e.g.,^56^). Beyond the amount of organics, the composition of organics also plays an important role in determining Mg signature, where, for example, different relative concentrations of peptides (i.e., short chains of amino acids) change Mg concentration^59^. In our results, the higher mean Mg/Ca concentrations in the low turbid reef may relate to the prevalence of certain types of peptides known to elevate Mg/Ca signal (e.g., simple hydrophilic peptides)^59^. Previous research has shown that for symbiotic hard corals the balance between heterotrophy versus autotrophy changes the relative abundance of amino acids in the organic matrix (e.g.,^60^). We propose a similar mechanism for *T. squamosa*, whereby environmental differences between sites change feeding activity of the host and symbionts (e.g. phytoplankton availability), in turn altering amino acid composition and Mg/Ca concentration. Similarly, juvenile tridacnids are known to show more reliance on heterotrophy than adults^61,62^, so ontogenetic changes related to feeding could explain lifetime trends in Mg/Ca previously recorded^38,44^. This work highlights the high-level role organics may play in relation to biogeochemical processes and how these parameters could be related to the feeding mechanism of giant clams.

In contrast to the association between Mg and organics, strontium substitutes for Ca^2+^ within the aragonite lattice and may predominantly enter the extrapallial fluid to the site of calcification through active transport mechanisms at the mantle epithelia, most notability Ca^2+^ ATPase (e.g.,^14^) (Fig. 5a). In both reef sites and all shell regions mapped, *T. squamosa* shows daily cyclicity in Sr/Ca. However, in relation to mean Sr/Ca values, we see a significant difference between sites during the wet season, with increased Sr/Ca concentration in the low turbid reef. Previous work has reported an inverse relationship with Sr and insolation in giant clams^41,63^, thought to reflect diurnal cyclicity related to the light enhanced calcification mechanism^41^. During light enhanced shell formation in the daytime, a lower Sr/Ca concentration is recorded because light dependent active transport of calcium into the extrapallial fluid by Ca^2+^ ATPase is enhanced, lowering the ratio of Sr/Ca^41^. This is in line with our findings not only on a daily scale, but also on a seasonal scale, where mean Sr/Ca is highest in the wet season, with the lowest levels of light^22^. However, it is known a strong biological control exists on Sr incorporation into the shell and individuals kept under continuous illumination^39^ or alternatively under a sunshade^40^ still exhibit Sr/Ca cyclicity. Overall, we find it is likely that external environment does play an indirect role in Sr/Ca concentrations; yet it is clear there is a strong physiological control, in line with previous studies^38–40,44^.

Aside from biological control, kinetic processes may also exert influence on elemental concentrations. Considering growth rates in both reef localities are similar within shell regions selected for EPMA, it is unlikely that there is a kinetic effect related to growth rate^64^. However, it is possible that other kinetic factors related to foreign ion partitioning may indirectly explain variations. For instance, variation in microstructure affects crystallographic orientation, which in turn impacts the amount of minor and trace elements bound to the mineral phase^65^. Potentially different mineral surface structures could show different affinities for elements that substantially vary across crystal faces^66^. Considering the microstructural and crystallographic variability of the IL in this study, it is noteworthy that microspatial differences in the abundance of elements could be constrained by crystal face symmetry and play a role in preferred element incorporation.

Our findings on the geochemistry and architecture of shells in this study have important implications for their biomechanical properties (e.g., hardness and elasticity), giving indication of the defensive capabilities and resilience of biogenic skeletons^11,34^. Overall, our results show higher crystallographic orderliness and significantly lower El/Ca ratios in the high turbid site, compared to the low turbid site. Higher crystallographic control (e.g.,^67^) and low Mg/Ca ratios (e.g.,^11^) are known to indicate superior mechanical properties and increased structural integrity of the carbonate skeleton, suggesting giant clam shells from the high turbid reef may be mechanically superior. These differences have been suggested to be driven by the amount of ACC and organic material, with higher concentrations linked to more Mg and decreased stiffness and hardness^11^. However, a previous study on symbiotic hard corals has shown that there is a strong relationship between skeletal embrittlement and high sedimentation rate, finding corals fractured under lower loads and were weaker with high turbidity^11^. It is important to highlight that shells from the high turbid reef may show compensatory responses for increased resilience to turbidity by forming biominerals with higher defensive capabilities, although more work is needed to understand how turbidity impacts embrittlement, hardness and stability of the carbonate skeleton in multiple species.

This study highlights plasticity in the biomineral formation of giant clam shells between high turbid and low turbid coral reefs in the Coral Triangle. Fine-scale modifications to the architecture and geochemistry of the inner shell layer are sensitive proxies of complex physiological adjustments in response to turbidity and a multitude of accompanying changing environmental factors on turbid reefs. The highlighted differences in microstructure, crystallographic co-orientation and microspatial distribution of Mg/Ca and Sr/Ca between reefs that may relate to energy partitioning and nutritional strategy are highly relevant in a rapidly changing ocean where turbidity is set to increase due to anthropogenic pressures. The results presented herein suggest that shell formation is modified potentially as a compensatory mechanism in turbid coral reefs and that they may be suitable habitats for *T. squamosa* and other mixotrophic marine corals and calcifiers under rapid environmental change and should be considered as important conservation refuge against bleaching and other stressors.

## Materials and methods

Our study was performed on four modern *T. squamosa* giant clam shells collected alive from Darvel Bay (4° 5356′ N, 118° 2646′ E), Sabah, Malaysia, Borneo (Fig. 1). The samples were collected on 16 April 2019 and 22, 23 February 2020 in 5–8 m water depth from Triangle reef (high turbid, sediment input from river source) and Baik reef (low turbid) (Supplementary Table 1, see 20 for more information). Climatic conditions within this region are controlled by the Indo-Australian monsoon system, divided into the southwest monsoon between May to September (dry season) and northeast monsoon from November to March (wet season)^68^. In our previous study^22^, we discerned that key environmental differences between these reefs are the light attenuation coefficient of downwelling irradiance at 490 nm (*K*_*d*_(490) (Supplementary Fig. 1), an indicator of turbidity within the water column, total suspended solids (Supplementary Table 1) and chlorophyll-*a* and, which are elevated year-round in Triangle compared to Baik. Lifespan was estimated by counting daily growth increments^22^ and ages ranged between approximately 1.86 and 7.23 years (Supplementary Table 1). These samples included a shell from each location that was estimated to be of a similar age (around 3 years) and analysis for both samples was carried out in ontogenetic years 1, 2 and 3 for SEM and EBSD and ontogenetic year 2 for EPMA for direct comparison. Shell lengths ranged from 203.96 to 362.62 cm and heights 143.50–215.08 cm (umbo to margin).

### SEM

Whole shell valves were cut longitudinally into around 2 cm thick slices along the axis of maximum growth with a circular diamond saw (Fig. 1a,b). Thin section (60 μm thickness) cut perpendicular to the direction of growth were prepared from slices and stuck to glass slides using epoxy resin. Sections were ground with 1000 grit sandpaper and polished with 0.3 μm alumina oxide. To prepare samples for SEM analysis, polished sections were etched with 0.5% HCL for 15 s to improve visibility of individual biomineral units and sputter coated with 20 nm gold–palladium (Au–Pd). A Zeiss Sigma HD field emission gun SEM (FEG–SEM) at the School of Earth and Environmental Sciences, Cardiff University was used under high vacuum with 10 kV accelerating voltage, aperture size 30 μm and working distance ~ 9.5 mm to obtain In-Lens Secondary Electron (SE) images of microstructure.

### EBSD

Sections used for SEM were subjected to several sequential mechanical grinding and polishing steps, with a final polish of 0.3 μm colloidal silica (70 rpm rotation, 20 min) using a Logitech PM5 automatic polisher. Afterward, samples were coated with a thin 3 nm layer of carbon. EBSD was carried out at the same distance (~ 4 mm inward from the hinge) along sections as the In-Lens SE images using the same SEM equipped with a Nordlys-2 EBSD detector. Diffraction patterns were collected at 0.5 μm step size in high current mode, 20 kV accelerating voltage, ~ 2.7 nA beam current and 60 μm aperture. Crystallographic preferred orientations (CPO) were indexed using Oxford Instruments AZtec 6.0 software. Parameters used for the indexing of the aragonite unit cell were the orthorhombic symmetry system from the OINA database with space group Pmcn *a* = 4.9614 Å, *b* = 7.9671 Å, *c* = 5.7404 Å and post-acquisition refinement carried out^66^. EBSD data is represented here as inverse pole figure maps (IPF), demonstrating crystal orientation relative to a reference frame, and corresponding contoured pole figures. The density distribution of contoured pole figures gives statistics on crystallographic co- orientation by multiple of uniform density (MUD) values, derived from pole figure maxima. A higher MUD value indicates a stronger co-orientation strength, which is indicative of a strong, highly organized fibre texture, while a random orientation of crystallographic distribution will have an MUD of 1 (e.g.,^54^).

### EPMA

Geochemical concentrations were measured on a JEOL 8530F field-emission electron microprobe equipped with five wavelength dispersive spectrometers (WDS) at the Electron Microbeam Suite at the University of Bristol, UK. Two *T. squamosa* thin sections from Baik and Triangle collected in 2019 used for SEM and EBSD (Supplementary Table 1) were fragmented with a diamond tipped scribe, and ground on a grinding machine equipped with a CUTROCK horizontal diamond wheel. Three 6 × 6 mm squares of each section were obtained that corresponded to periods of growth from (1) the OL, (2) the IL wet season (~ December 2017) and (3) the IL dry season (~ August 2018). Squares were coated with 10 nm of silver (Ag) to minimize beam damage and ensure sample stability. 100 × 100 μm maps for Mg (TAP and TAPH crystals) and Sr (TAP crystal) were acquired at 15 kV and 100 nA with a pixel size of 0.9 μm and 500 ms dwell time. Spot measurements (3 μm beam diameter) were acquired at 15 kV and 100 nA at 5 μm intervals. Measurements for Ca (PETH crystal) were acquired with a 3 μm beam diameter and 5 μm interval at 15 kV and 5 nA. The instrument was calibrated with the following internal standards: Iceland Spar Calcite (ICE) for Ca, MgO for Mg and SrSO4 for Sr. Maps were quantified using CalcImage in Probe for EPMA software package (http://www.probesoftware.com/).

To generate Mg/Ca and Sr/Ca maps, shell Mg and Sr concentrations were normalized to Ca in Image J 1.53 software^57^ and pixels with Ca wt% below 35 masked. Any negative Mg values were substituted with half of the minimum value above zero^58^. The detection limit for Mg for an average of 4 pixels was 0.009 wt% (or 1 pixel = 0.017 wt%) to 2 SD. If wt% Ca = 40, analytical uncertainty at 2 mmol/mol Mg/Ca (close to average map values) for an average of 4 pixels is ~ 11%; this drops to ~ 5% when averaging 16 pixels. The detection limit for Sr for an average of 4 pixels is 0.037 wt% (or 1 pixel = 0.073 wt%) to 2 SD. If wt% Ca = 40, analytical uncertainty at 3 mmol/mol Sr/Ca (close to average map values) for an average of 4 pixels is ~ 10%; this drops to ~ 5% if 16 pixels are averaged. To achieve similar uncertainty at lower ratios, more pixels need to be averaged. For example, for Mg/Ca analytical uncertainty at 1 mmol/mol for an average of 4 pixels is ~ 21% and this drops to 10% if 16 pixels are averaged.

To test for significant differences between the means of Sr/Ca and Mg/Ca from different regions within shells corresponding to different growing seasons (i.e., wet and dry season) and between shells collected from the different reef localities, a one-way analysis of variance (ANOVA) test was conducted in the statistical software R^69^ (Supplementary Table 2). Data was assessed for normality and homogeneity of variances using Shapiro-Wilk’s test, QQ (quantile–quantile) plots of standardized residuals and Bartlett’s test. Assumptions of ANOVA were satisfied for Sr/Ca, but for Mg/Ca the nonparametric Kruskal–Wallis test was used (Supplementary Table 3). Differences between groups were investigated using either a post-hoc Tukey HSD test (ANOVA) or Dunn’s multiple comparisons test (Kruskal–Wallis).

## Electronic supplementary material

Below is the link to the electronic supplementary material.

Supplementary Material 1

## Data Availability

The original contributions presented in the study are included in the article and Supplementary Material. All Element-to-Calcium ratios associated with the article can also be found in the Mendeley data repository under DOI: 10.17632/w93czv65vg.2. Any further inquiries can be directed to the corresponding author.
