# Supplementary material for: Crystallographic and geochemical responses of giant clams on turbid reefs
Source: Sci Rep. 2025 Mar 2;15:7365. doi: 10.1038/s41598-025-90614-y (PMC11873261; doi:10.1038/s41598-025-90614-y)
Supplement: Supplementary file 1 — Supplementary Material 1 [file 41598_2025_90614_MOESM1_ESM.docx]

**Supplementary information**

**Giant clams modify crystallographic and geochemical pathways of shell formation in response to turbidity**

Kimberley Mills^1*^, Sindia Sosdian^1^, Duncan D. Muir^1^, Eleanor H. John^1^, Nadia Santodomingo^2^, Kenneth Johnson^2^, Ben Buse^3^, Zarinah Waheed^4^

**Supplementary Table 1:** List of four studied samples of *Tridacna squamosa* from Darvel Bay. BA = Baik; TR = Triangle. Estimated age calculated from daily growth increments in Mills et al. (2023). Collection status: L = shells collected alive in either 2019 or 2020. SEM = scanning electron microscopy; EBSD = electron backscatter diffraction; EPMA = electron probe microanalysis. Annual mean *K_d_*(490) is given for 2019–2020 and annual mean total suspended solids (TSS) content collected from sites are in milligrams per litre (mg/L).

| Site | Species | Sample ID | Collection Status | Collection Depth (m) | Estimated Age | Mean growth (mm/yr) | Method | Annual mean *K_d_*(490) | Total suspended solids (TSS) |
| --- | --- | --- | --- | --- | --- | --- | --- | --- | --- |
| TR | TS | SSct | L 2019 | 5 | 3.40 | 5.24 ± 1.46 | SEM, EBSD, EPMA | 0.30 | 7.66 |
| BA | TS | SSbaik | L 2019 | 7 | 3.10 | 5.20 ± 2.48 | SEM, EBSD, EPMA | 0.09 | 4.58 |
| TR | TS | ZW156 | L 2020 | 5 | 1.86 | 8.13 ± 3.37 | SEM, EBSD | 0.30 | 7.66 |
| BA | TS | NS207 | L 2020 | 8 | 7.23 | 4.21 ± 1.95 | SEM | 0.09 | 4.58 |

**Supplementary Figure 1.** Monthly average *K_d_*(490) for Baik and Triangle reefs in Darvel Bay from 2017–2020 derived from NOAA Visible Infrared Imaging Radiometer Suite at 0.05° x 0.05° resolution.


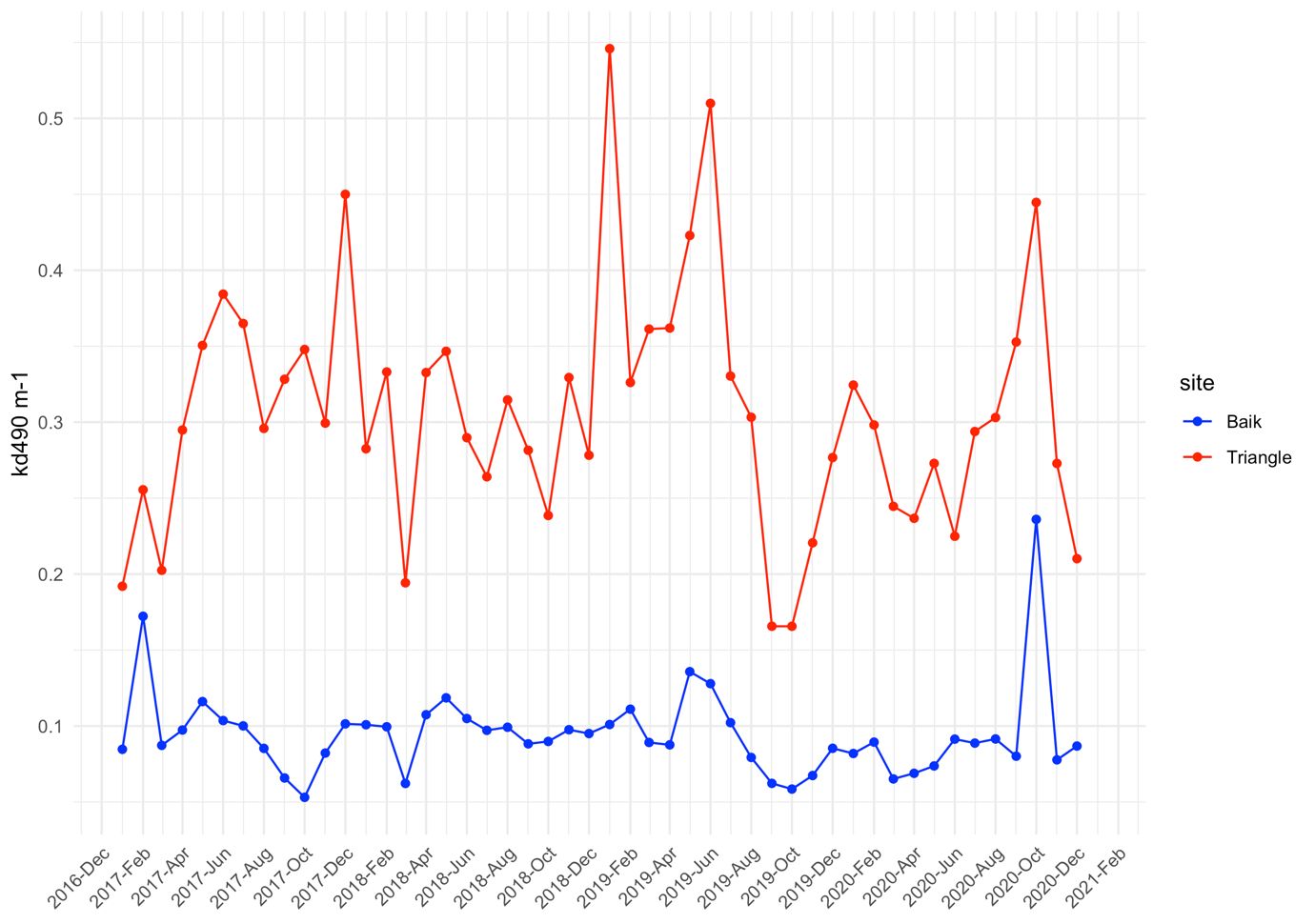


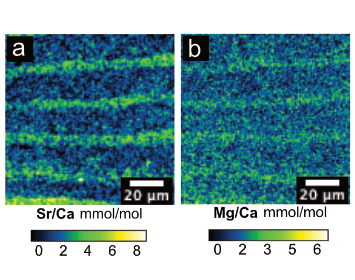


**Supplementary Figure 2.** Sub-daily resolved EPMA maps for (a) Sr/Ca and (b) Mg/Ca in a region of the outer shell layer in the low turbid giant clam (Baik) shell. Color bars below maps show concentrations of Element-to-calcium rations in mmol/mol.

**Supplementary Table 2:** Post hoc Tukey HSD test results for one-way ANOVA (*F*(3) = 43.58) for Sr/Ca between shells from the Triangle and Baik reefs in the wet and dry seasons. Upper matrix reports *P*-values (significant values of <0.05 in bold). Lower matrix is the difference.

|  | SSBAWS | SSBADS | SSCTWS | SSCTDS |
| --- | --- | --- | --- | --- |
| SSBAWS |  | **<0.01** | **<0.01** | **<0.01** |
| SSBADS | 0.86 |  | 0.33 | 0.90 |
| SSCTWS | -0.62 | 0.24 |  | 0.12 |
| SSCTDS | -0.80 | 0.06 | 0.18 |  |

**Supplementary Table 3:** Pairwise comparison of Mg/Ca from Kruskal-Wallis test (χ²(3) = 40.7) between shells from the Triangle and Baik reefs in the wet and dry seasons using Dunn’s multiple comparisons test. *P*-values (significant values of <0.05 in bold) are adjusted with the Benjamini-Hochberg (BH) method.

| Comparison | *P*-value (BH adjusted) |
| --- | --- |
| SSBAWS - SSBADS | **0.02** |
| SSCTDS-SSBADS | **<0.01** |
| SSCTWS-SSBADS | **<0.01** |
| SSCTDS-SSBAWS | **0.03** |
| SSCTWS-SSBAWS | **<0.01** |
| SSCTWS-SSCTDS | 0.16 |

**References**

Mills, K. et al., Growth responses of mixotrophic giant clams on nearshore turbid coral reefs. *Coral Reefs* **42**, 593–608 (2023).
